# A Combined SIRT5 Activation and SIRT3 Inhibition Prevents Breast Cancer Spheroids Growth by Reducing HIF-1α and Mitophagy

**DOI:** 10.3390/ph19010023

**Published:** 2025-12-22

**Authors:** Federica Barreca, Michele Aventaggiato, Mario Cristina, Luigi Sansone, Manuel Belli, Maria Beatrice Lista, Gaia Francisci, Sergio Valente, Dante Rotili, Antonello Mai, Matteo Antonio Russo, Marco Tafani

**Affiliations:** 1 Department of Experimental Medicine, Sapienza University of Rome, 00161 Rome, Italy; federica.barreca@uniroma1.it (F.B.); mariabeatrice.lista@uniroma1.it (M.B.L.); gaia.francisci@uniroma1.it (G.F.); 2 Department of Life Sciences, Health, and Health Professions, Link Campus University, 00165 Rome, Italy; 3 Laboratory of Molecular, Cellular and Ultrastructural Pathology, IRCCS San Raffaele Roma, 00166 Rome, Italy; mario.cristina@sanraffaele.it (M.C.); luigi.sansone@sanraffaele.it (L.S.); manuel.belli@uniroma5.it (M.B.); matteoantoniorusso44@gmail.com (M.A.R.); 4 Department of Human Sciences and Promotion of the Quality of Life, San Raffaele Roma Open University, 00166 Rome, Italy; 5 Department of Molecular Medicine, Sapienza University of Rome, 00161 Rome, Italy; 6 Department of Drug Chemistry and Technologies, Sapienza University of Rome, 00185 Rome, Italy; sergio.valente@uniroma1.it (S.V.); antonello.mai@uniroma1.it (A.M.); 7 Department of Science, Roma Tre University, Viale Guglielmo Marconi, 446, 00146 Rome, Italy; dante.rotili@uniroma3.it

**Keywords:** sirtuins, glutamine metabolism, autophagy, mitophagy, triple negative breast cancer

## Abstract

**Background/Objectives:** Metabolic reprogramming is an essential feature of tumors. Mitochondrial sirtuins SIRT3 and SIRT5 differently regulate glutamine metabolism with SIRT5 inhibiting glutaminase (GLS) and SIRT3 increasing glutamate dehydrogenase (GDH). Considering the important and interconnected role of glutamine, SIRT3 and SIRT5 for cancer growth and progression, our hypothesis is that a simultaneous modulation of SIRT3 and SIRT5 could represent a valid anti-tumoral strategy. **Methods:** wt and GLS1-silenced triple negative breast cancer spheroids were treated with 3-TYP, a selective SIRT3 inhibitor, and with MC3138, a new selective SIRT5 activator, both alone and in combination. The effects of such treatments on hypoxia, autophagy and mitophagy markers were determined by immunofluorescence and Western blot. Mitochondria morphology was studied by transmission electron microscopy (TEM) and mitochondrial ROS production by confocal analysis. **Results:** We observed that 3-TYP+MC3138 treatment decreased the size of spheroids by affecting HIF-1α, c-Myc, glutamine transporter SLC1A5 and autophagy (LC3II) and mitophagy (BNIP3) markers. Moreover, such treatments altered the morphology and conformation of the mitochondria. Finally, we also documented an increase in mitochondria reactive oxygen species (mtROS). **Conclusions:** The combined inhibition of SIRT3 and activation of SIRT5 greatly reduces the size of spheroids through the inhibition of hypoxic response, which is then followed by the alteration of the autophagic and mitophagic process and the toxic accumulation of mitochondrial ROS, representing a new anti-tumoral strategy.

## 1. Introduction

The most frequently diagnosed cancer in women is breast cancer [1]. This type of cancer is very heterogeneous in course, prognosis and sensitivity to therapy. Triple negative breast cancers (TNBCs) lack estrogen receptors (ERs), progesterone receptors (PRs), and oncoprotein HER-2/neu (human epidermal growth factor receptor 2), have a higher recurrence rate, greater metastatic potential and lower overall survival rate [1,2]. The main therapeutic strategies consist of a combined treatment involving surgical removal of tumor and metastasis, topical radiotherapy and chemotherapy in neoadjuvant and/or adjuvant regimens [3]. Cancer cells alter their metabolism to increase their proliferative capacity [4]. Under nutrient deficiency and hypoxia, cancer cells shift their metabolism from using glucose to using glutamine. In triple negative breast cancer cells, glutaminase (GLS) is overexpressed, and this is associated with high-grade metastatic breast cancer [5]. Several studies have shown that, in TNBC more than in luminal-type breast cancers, a reduction in the activity of GLS is accompanied by a reduction in tumor proliferation and progression [6].

Sirtuins have an important role in the different steps of cancer development, acting both as tumor promoters and inhibitors depending on the tumor type and stage [7,8]. There are seven mammalian sirtuins (SIRT1-SIRT7), with mono-ADP-ribosyltransferase and deacylase activity, and they were initially described as class III histone deacetylases [9]. These seven sirtuins are expressed in different cellular compartments: SIRT1, SIRT6 and SIRT7 in the nucleus, SIRT2 in cytoplasm, while SIRT3, SIRT4 and SIRT5 are usually located in mitochondria. SIRT1 can also be found in the cytoplasm, and SIRT3 can be found in the nucleus [10]. Sirtuins are involved in cell metabolism through epigenetic modulation, catalysis or by the regulation of a plethora of signaling pathways in different types of cancer [9]. Mitochondrial sirtuins, SIRT3, SIRT4, and SIRT5, are emerging as key regulators of cancer metabolism. SIRT3, the major mitochondrial sirtuin, governs mitochondrial function and cellular bioenergetics, impacting fatty acid oxidation, amino acid metabolism and the tricarboxylic acid cycle [11]. SIRT3 can be either a tumor-inducer or a suppressor. In some types of cancer, such as prostate or hepatocellular carcinoma, the decrease in SIRT3 expression is linked to an increase in tumorigenesis and tumor metabolism with the increase in reactive oxygen species (ROS) and activation of the hypoxia inducible factor-1α (HIF-1α) [12]. Meanwhile, in head/neck tumors and melanomas, the increased expression of SIRT3 promotes carcinogenesis and resistance to apoptotic stimuli, as well as resistance to radiotherapy through the nuclear factor kappa-light-chain-enhancer of activated B cells (NF-kB) pathway [13]. SIRT5 plays an essential role in the acetylation/succinylation state of different substrates. This sirtuin is involved in the urea cycle by deacetylating and activating carbamoyl phosphate synthetase 1 (CPS1) to catalyze the elimination of ammonia in the hepatocytes [14]. In addition, SIRT5 controls glutamine metabolism by regulating glutaminase activity [15]. Like SIRT3, SIRT5 can also function as tumor promoter or tumor suppressor. For example, studies have shown that SIRT5 expression is down-regulated in squamous cell carcinoma and endometrial carcinoma [14]. Interestingly, both SIRT3 and SIRT5 control glutamine metabolism. In fact, SIRT3 deacetylates and activates acetyl-CoA synthetase 2 and Glutamate dehydrogenase (GDH), promoting glutamine oxidation [16], while SIRT5 controls glutamine metabolism by desuccinylating and inhibiting GLS [15]. In this way, they tightly control the formation of α-ketoglutarate from glutamine to replenish the tricarboxylic acid cycle (TCA). Curiously, there are very few studies on the crosstalk between sirtuins in general and mitochondrial sirtuins in particular. We have previously shown a crosstalk between SIRT1 and SIRT3 in breast cancer cells [17]. Similarly, a crosstalk between SIRT1, SIRT3 and SIRT5 as part of a mitochondrial–nuclear crosstalk has been proposed and studied in osteosarcoma and mesothelioma cells [18,19]. For this reason and because of the development of new and more specific sirtuins modulators, we believe that it is now possible to better dissect the complexity of crosstalk among the seven sirtuins. To do that, we decided to use 3D cellular models. In fact, breast cancer spheroids are more aggressive and resistant to treatment than monolayer cells [20,21]. Moreover, spheroids obtained from cancer cells create a hypoxic core region, an acidic extracellular pH and a modified cellular metabolism. The presence of hypoxia makes the tumor resistant to conventional therapies and increases its metastatic potential [22]. There is little information on the effects of mitochondrial sirtuins modulation on spheroids of triple negative MDA-MB-231 breast cancer cells. Therefore, the present work used spheroids of wild type and GLS1-silenced MDA-MB-231 treated with 3-TYP and MC3138, in order to determine morphological and molecular effects on these 3D structures. Our results show that these treatments result in a reduction in the size of the spheroids, a decrease in HIF-1α expression and mitophagy and an increase in ROS.

## 2. Results

### 2.1. SIRT3 Inhibition and SIRT5 Activation Reduce the Growth of MDA-MB-231 Spheroids

Mitochondrial sirtuins play an important role in controlling the health of these organelles [11]. The expression and activity of mitochondrial sirtuins is often modulated in cancer cells to sustain their rapid growth and survival [14]. Glutamine metabolism has a central role in cancer cell growth and survival [23]. We have previously shown that, in TNBC, the mitochondrial SIRT5 has an important role in glutamine metabolism by regulating glutaminase, the enzyme involved in the first step of this pathway [15]. On the other hand, SIRT3 can also modulate glutamine metabolism by regulating glutamate dehydrogenase, the enzyme involved in the second step [24]. Therefore, our hypothesis is that a simultaneous modulation of SIRT3 and SIRT5 could represent a valid anti-tumoral strategy by reprogramming glutamine metabolism.

**Figure 1:**
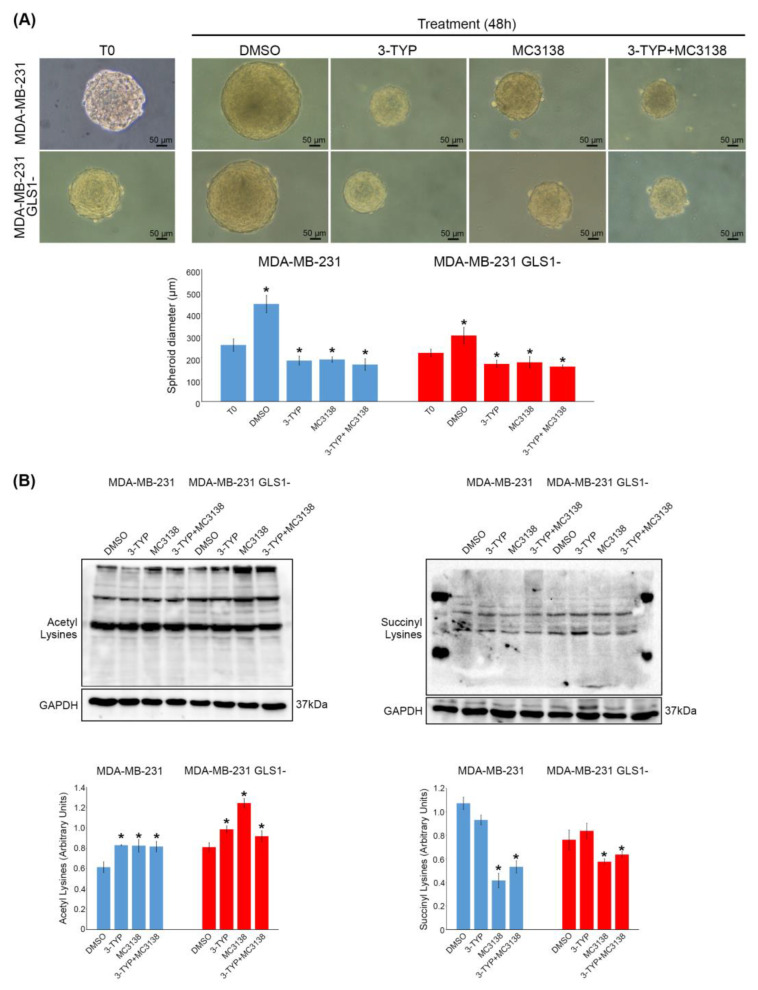
3-TYP and MC3138 treatments reduce the growth of spheroids. (**A**) Spheroids of MDA-MB-231 wt and GLS1- cells were treated for 48 h with 3-TYP, MC3138 and 3-TYP plus MC3138 as described under Materials and Methods. Representative images of the spheroids were taken with a phase contrast microscopy. Mean spheroids diameter (µm) indicated in the graphs was obtained by measuring five spheroids from three separate experiments. (**B**) Spheroids treated with DMSO (control) or with 3-TYP, MC3138 and 3-TYP plus MC3138 were lysed and protein extracted as described under Materials and Methods. Lysines acetylation and succinylation was determined by Western blot. GAPDH was used as loading control. Densitometric analysis of the gels was performed as described under Materials and Methods, and the results graphed below the blots. * Significantly different from DMSO-treated cells, *p* < 0.05. Experiments were repeated three times. Statistical analysis was performed by Student’s *t*-test.

Triple negative breast cancer cells MDA-MB-231 were treated with previously validated SIRT3 inhibitor (3-TYP) and SIRT5 activator (MC3138) [15,25,26]. Moving on from these previous studies, we determined the concentration of MC3138 and 3-TYP through vitality and clonogenic assays. The results are shown in Supplementary Figures S1 and S2. We observed a 15% cell death with MC3138 and a very low cell killing activity with 3-TYP, even at higher concentrations (Figures S1A and S2A). On the contrary, both molecules showed a strong inhibition of clonogenic ability of MDA-MB-231 cells, even at low concentrations (Figures S1B and S2B). For this reason, we relied on the clonogenic assay and determined the concentrations used in our experiments to be 50 µM for MC3138 and 25 nM for 3-TYP, where we had a 50% reduction in clones (Figures S1B and S2B). The same treatments were then applied to our previously established MDA-MB-231 GLS1- cell line [15]. As shown in Supplementary Figure S3, MDA-MB-231 wt and GLS1- cells were treated for 48h with 50 µM of MC3138, 25 nM of 3-TYP or a combination of both. Vitality and clonogenic assays were carried out at the end of the treatments. Again, our results show that there was a 15% reduction in wt and GLS1- cell vitality only in the presence of MC3138 treatment (Figure S3). The clonogenic assay showed a decrease in colonies formation with all the treatments (Figure S4). However, the strongest inhibition was obtained with MC3138 plus 3-TYP treatment (Figure S4). Interestingly, the MDA-MB-231 GLS1- cells had a lower number of colonies (Figure S4). However, there was still an inhibitory effect of MC3138 and 3-TYP (Figure S4). Importantly, the same MC3138 and 3-TYP concentrations did not cause cell death in primary fibroblasts, as shown in Supplementary Figure S5. Taking into consideration the effects of MC3138 on cell death and of MC3138 and 3-TYP on clones formation, and considering the known effect of SIRT3 and SIRT5 on glutamine metabolism, we performed the same treatments on spheroids of wt and GLS1- MDA-MB-231 cells. Compared to untreated or DMSO-treated controls, treatments of 3-TYP, MC3138 and 3-TYP+MC3138 all reduced the size of spheroids (Figure 1A).

However, such a reduction was more evident with the combined treatment, where we observed a dark necrotic center and several detached cells from the spheroids (Figure 1A). Interestingly, MDA-MB-231 GLS1- cells formed smaller spheroids than wt cells (Figure 1A). We also measured the size of the spheroids, confirming a reduction upon single or combined treatment (Figure 1A). In fact, 48h of the combined treatment significantly reduced spheroids’ size from 443 µm of dimethyl sulfoxide (DMSO) to the 168 µm of 3-TYP+MC3138 in wt cells and from 300 µm of DMSO to 158 µm of 3-TYP+MC3138 in GLS1- cells (Figure 1).

Since we are inhibiting the SIRT3 deacetylase activity with 3-TYP and activating the desuccinylase activity of SIRT5 with MC3138, we aimed to determine the global acetylation and succinylation levels in our untreated or treated spheroids (Figure 1B). As expected, 3-TYP treatment increased acetylated lysines (Figure 1B left graph). Similarly, MC3138 decreased succinylated lysines (Figure 1B right graph). Interestingly, we observed that acetylated lysines also increased with MC3138, an event that is not due to SIRT3 modulation by MC3138, since we have extensively documented the specificity of this activator [15,26]. As also commented in the Discussion, our explanation is that activation of SIRT5 desuccinylating activity by MC3138 leaves “free” lysines that are rapidly acetylated to maintain a dynamic homeostatic balance in the mitochondria. On the contrary, we do not observe a decrease in succinylated lysines with 3-TYP because inhibition of SIRT3 maintains those lysines acetylated (Figure 1B). Finally, as expected, in the combined treatment we have both an increase in acetylated and a decrease in succinylated lysines (Figure 1B).

### 2.2. SIRT3 Inhibition and SIRT5 Activation Alter the Expression of Autophagy and Mitophagy Markers

**Figure 2:**
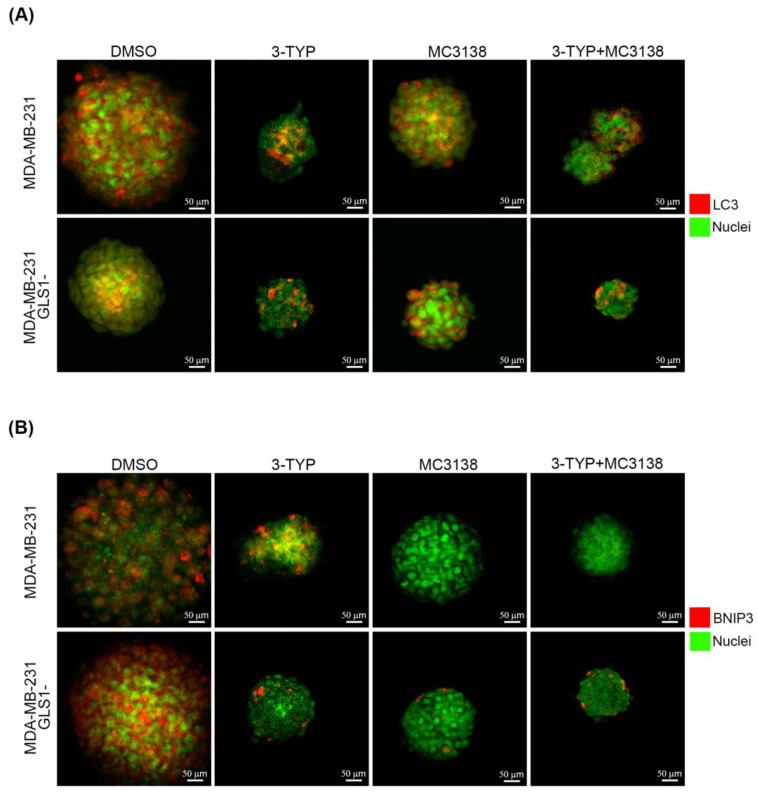
Reduced LC3 and BNIP3 fluorescence in spheroids after 3-TYP and MC3138 treatments. Spheroids of MDA-MB-231 wt and GLS1- cells were treated for 48h with 3-TYP, MC3138 and 3-TYP plus MC3138 as described under Materials and Methods. At the end of the treatments, spheroids were fixed and permeabilized. LC3 (**A**) and BNIP3 (**B**) fluorescence was determined as described under Materials and Methods. Nuclei were stained with SYTO 16 green fluorescent nucleic acid stain. Experiments were repeated three times.

We have previously demonstrated the effect of SIRT5 activation through MC3138 on glutamine metabolism in triple negative breast cancer cells [15]. In particular, using a 2D model of MDA-MB-231 cells, we have shown that MC3138 reduces glutamine metabolism and ammonia production, an effect that, in turn, reduces the protective autophagic process [15]. Considering these data, we investigated the effects on autophagy and mitophagy of SIRT5 activation with MC3138 and SIRT3 inhibition with 3-TYP on a 3D model of MDA-MB-231 spheroids. The autophagic marker microtubule-associated protein 1A/1B-light chain 3 (LC3) was clearly expressed in the control DMSO spheroids of wt cells. Single treatment with 3-TYP or MC3138 reduced both LC3 expression and spheroid size (Figure 2A).

**Figure 3:**
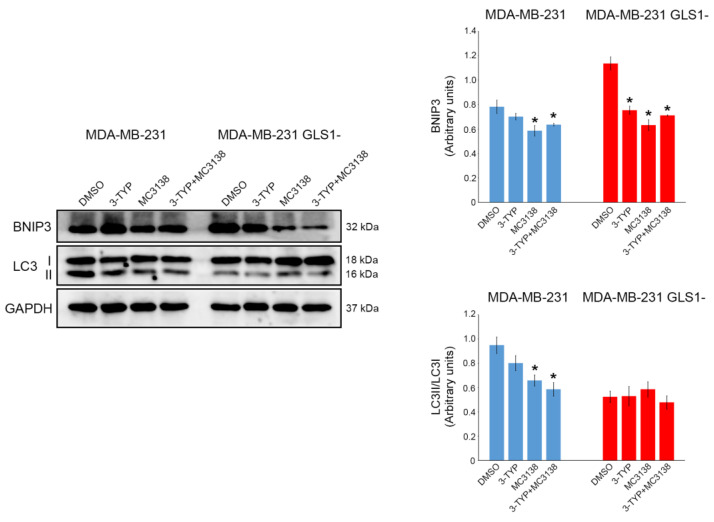
Reduced LC3 and BNIP3 expression in spheroids after 3-TYP and MC3138 treatments. Spheroids treated with DMSO (control) or with 3-TYP, MC3138 and 3-TYP plus MC3138 were lysed and protein extracted as described under Materials and Methods. LC3 and BNIP3 expression was determined by Western blot. GAPDH was used as loading control. Densitometric analysis of the gels was performed as described under Materials and Methods, and the results graphed below the blots. * Significantly different from DMSO-treated cells, *p* < 0.05. Experiments were repeated three times. Statistical analysis was performed by Student’s *t*-test.

Importantly, the combination of 3-TYP and MC3138 treatment greatly reduced the size of the spheroids, as well as LC3 expression (Figure 2A). In the case of spheroids of GLS1- cells, we observed that LC3 expression was lower and limited to the center of the spheroid (Figure 2A). Moreover, single or combined treatments with 3-TYP and MC3138 did not seem to impact LC3 expression, while reducing the size of the spheroids (Figure 2A). The mitophagic marker Bcl-2 interacting protein 3 (BNIP3) was expressed in spheroids of wt control DMSO cells but was almost undetectable after treatment with MC3138 or 3-TYP+MC3138 (Figure 2B). Similar results were obtained on spheroids of GLS1- cells, where, however, BNIP3 expression was higher in the DMSO control and lower but still present in the spheroids subjected to single or combined 3-TYP and MC3138 treatment (Figure 2B). These results were confirmed and quantified by Western blot analysis of the spheroids. In fact, a statistically significant decrease in LC3 II expression was observed in wt spheroids after MC3138 or 3-TYP+MC3138 treatment (Figure 3).

Compared to wt spheroids, GLS1- spheroids showed lower LC3 II expression, with no changes after single or combined 3-TYP and MC3138 treatments (Figure 3). Similarly to the results seen in Figure 2B, BNIP3 expression was higher in DMSO GLS1- than in wt spheroids and decreased after MC3138 or 3-TYP+MC3138 treatment in wt spheroids and after 3-TYP, MC3138 and 3-TYP+MC3138 in GLS1- cells (Figure 3). Similar results were obtained by inhibiting GLS1 with 10 µM BPTES. In fact, we observed a reduction in BNIP3 and LC3 expression only in the presence of MC3138 plus 3-TYP (Figure S6A).

These results evidence a decrease in autophagy and mitophagy markers following the combined treatment, thereby leaving MDA-MB-231 cells with unremoved dysfunctional mitochondria.

### 2.3. SIRT3 Inhibition and SIRT5 Activation Alter the Morphology of MDA-MB-231 Spheroids

**Figure 4:**
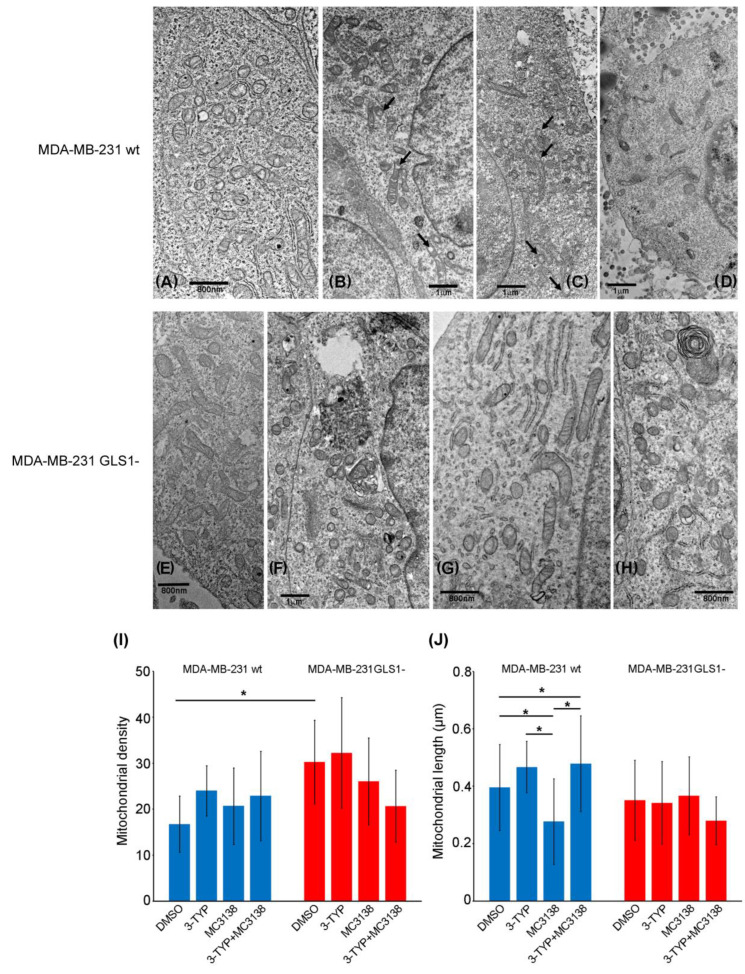
TEM analysis of mitochondrial changes in spheroids treated with 3-TYP and MC3138. Mitochondrial changes in DMSO, 3-TYP, MC3138 and 3-TYP plus MC3138 spheroids from MDA-MB-231 wt (**A**–**D**) and GLS1- (**E**–**H**) cells were investigated after fixing and staining by TEM, as described under Materials and Methods. Black arrows indicate an increase in intermembrane space in the mitochondria. Mitochondria numerical density (**I**) and length (**J**) were evaluated using at least 15 TEM images at the same magnification (2500×) on at least five equatorial sections (distance between the sections: 3–4 µm) and analyzed as described under Materials and Methods. *, *p* < 0.05. Experiments were repeated three times. Statistical analysis was performed by using one-way ANOVA with Tukey honest significant difference (HSD) tests for post hoc analysis.

Morphological and ultrastructural analysis was made through transmission electron microscopy of wt and GLS1- spheroids of MDA-MB-231 cells. Given the influence of our treatments on mitophagy markers, we focused our attention on the mitochondria population of the control and treated spheroids. In wt control spheroids, we observed numerous mitochondria in the orthodox state as defined by Hackenbrock [27]. Also, the cristae and the matrix were of normal size and typical of orthodox mitochondria (Figure 4A).

Treatment with the SIRT3 inhibitor 3-TYP resulted in an increase in mitochondria in the condensed state with dilated or vesiculated cristae (Figure 4B black arrows). On the other hand, SIRT5 activation with MC3138 changed mitochondria conformation to an intermediate orthodox-condensed state. We also observed an increase in the intermembrane space, as pointed by the black arrows in Figure 4C. Finally, the combined 3-TYP+MC3138 treatment resulted in a clear cellular stress with membrane shedding and loss of cell–cell contacts (Figure 4D). Mitochondria were in the condensed state (Figure 4D). Untreated control GLS1-silenced spheroids presented cells with mitochondria in the intermediate orthodox-condensed state (Figure 4E). Spheroids treated with 3-TYP had condensed mitochondria with few cristae (Figure 4F). Spheroids treated with MC3138 had mitochondria in the intermediate orthodox-condensed conformation (Figure 4G). Finally, spheroids treated with 3-TYP+MC3138 showed condensed mitochondria (Figure 4H). TEM images were also used to measure the density and the size of the mitochondria. These results are summarized in the graphs of Figure 4. In MDA-MB-231 wt spheroids the mitochondria density had a tendency to increase after the treatments, even if this result did not reach a statistical significance (Figure 4I). Interestingly, the mitochondrial density was significantly higher in the untreated or 3-TYP-treated GLS1- spheroids, to then decrease with MC3138 and 3-TYP plus MC3138 (Figure 4I). On the other hand, the length of the mitochondria was higher in the wt than in the GLS1-spheroids (Figure 4J). The length of the mitochondria decreased after SIRT5 activation with MC3138 and increased after SIRT3 inhibition with 3-TYP (Figure J). The increase in length was also observed with the double treatment 3-TYP+MC3138 (Figure 4J). No change in mitochondrial length was observed in the GLS1- spheroids, even in the presence of the different treatments (Figure 4J), suggesting a role for the glutamine metabolism in regulating mitochondrial size.

### 2.4. SIRT3 Inhibition and SIRT5 Activation Influence HIF-1α, c-Myc and SLC1A5 Expression

**Figure 5:**
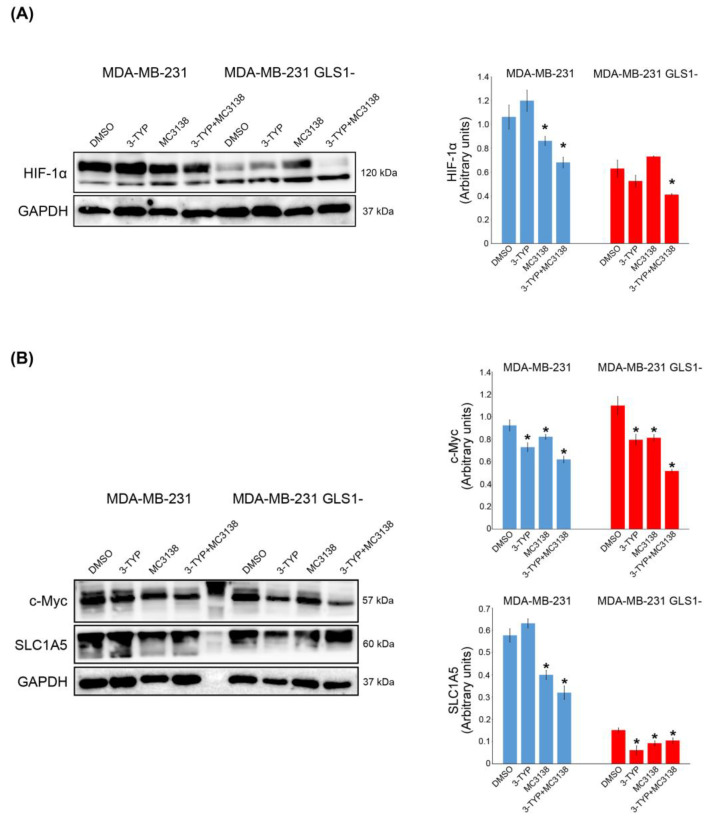
Reduction in HIF-1α, c-Myc and SLC1A5 in spheroids treated with 3-TYP and MC3138. Spheroids treated for 48 h with DMSO (control) or with 3-TYP, MC3138 and 3-TYP plus MC3138 were lysed and protein extracted as described under Materials and Methods. (**A**) HIF-1 α, expression was determined by Western blot. (**B**) c-Myc and SLC1A5 expression was determined by Western blot. GAPDH was used as loading control. Densitometric analysis of the gels was performed as described under Materials and Methods, and the results graphed close to the blots. * Significantly different from DMSO-treated cells. *p* < 0.05. Experiments were repeated three times. Statistical analysis was performed by Student’s *t*-test.

HIF-1α and Myelocytomatosis viral oncogene homolog (c-Myc) represent two genes with a central role in regulating cancer cell metabolism in general and glutamine metabolism in particular [28,29]. Both HIF-1α and c-Myc regulate the transcription of genes involved in glutamine transport and utilization, thereby increasing cancer cell growth and survival [30]. In fact, c-Myc and glutamine regulate transcription of the glutamine transporter solute carrier family 1 member 5 (SLC1A5) [31,32]. In addition, HIF-1α also regulates transcription of the mitophagy marker BNIP3 to sustain the removal of dysfunctional mitochondria from cancer cells [33]. The expression on HIF-1α decreased after treating MDA-MB-231 wt and GLS1- spheroids with 3-TYP and MC3138 either alone or in combination (Figure 5A).

Again, the effect was more evident with the combined treatment (Figure 5A). Similarly, the expression of c-Myc decreased after single or combined 3-TYP and MC3138 treatments (Figure 5B). Also, in this case, employing BPTES to inhibit GLS1 produced results similar to GLS1 silencing, in which HIF-1α and c-Myc reduction was observed only in the presence of MC3138 plus 3-TYP (Figure S6B). The glutamine transporter SLC1A5 followed c-Myc decrease with MC3138 or 3-TYP+MC3138 in wt MDA-MB-231 spheroids (Figure 5B lower blot). In MDA-MB-231 GLS1- spheroids we documented a basal low expression of SLC1A5, with a further decrease after the treatments (Figure 5B lower blot), an effect probably linked to the reduced glutamine metabolism.

### 2.5. SIRT3 Inhibition and SIRT5 Activation Reduce Lysosomal Degradation of Mitochondria and Increase Mitochondrial ROS Production

**Figure 6:**
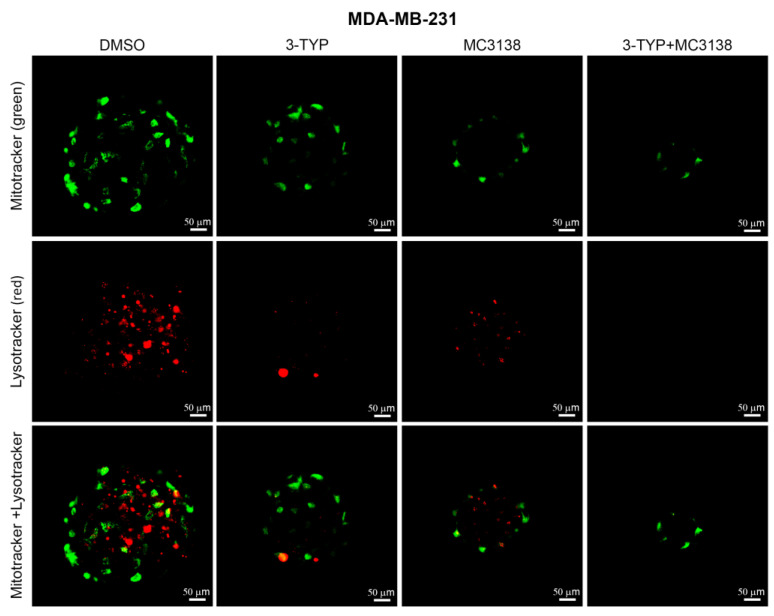
Reduction in mitochondria and lysosomes co-localization in MDA-MB-231 wt spheroids treated with 3-TYP and MC3138. Spheroids were either treated with DMSO or with 3-TYP, MC3138 or 3-TYP plus MC3138 for 48 h. At the end of the treatments, spheroids were incubated with Lysotracker Red to stain lysosomes and then with Mitotracker green to stain mitochondria as described in Materials and Methods. Fluorescence from lysosomes and mitochondria was visualized through a confocal microscope. The yellow fluorescence observed represents the lysosomes–mitochondria co-localization.

To confirm that the damage produced by our treatments was also due to the inhibition of autophagy and mitophagy with accumulation of dysfunctional mitochondria, we stained mitochondria and lysosomes using Mitotracker (green) and Lysotracker (red). As shown in Figure 6, in wt MDA-MB-231 spheroids, there is a mitochondria–lysosomes co-localization represented by the yellow fluorescence in DMSO control.

**Figure 7:**
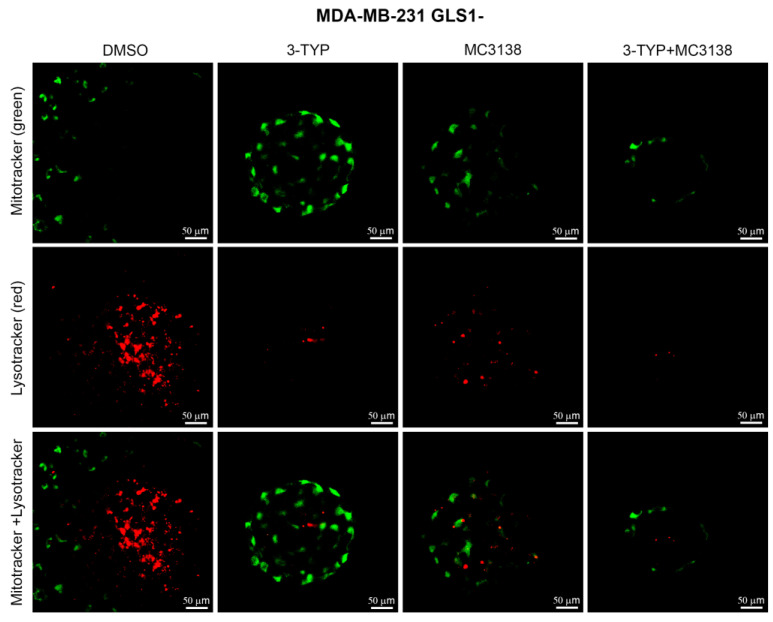
Reduction in mitochondria and lysosomes co-localization in MDA-MB-231 GLS1- spheroids treated with 3-TYP and MC3138. Spheroids were either treated with DMSO or with 3-TYP, MC3138 or 3-TYP plus MC3138 for 48 h. At the end of the treatments, spheroids were incubated with Lysotracker Red to stain lysosomes and then with mitotracker green to stain mitochondria as described in Materials and Methods. Fluorescence from lysosomes and mitochondria was visualized through a confocal microscope. The absence of yellow fluorescence is noteworthy, as it indicates the absence of a lysosomes–mitochondria co-localization.

Such co-localization was reduced but still present after 3-TYP and MC3138 single treatment, whereas it was completely abolished with the combined treatment, where only mitochondria green staining was retained (Figure 6). Again, this study also confirmed the reduction in size of the spheroids after the combined treatment (Figure 6). On the other hand, the mitochondria–lysosomes co-localization was strongly reduced in DMSO control of MDA-MB-231 GLS1- spheroids (Figure 7). Combined 3-TYP+MC3138 treatment completely abolished such co-localization (Figure 7).

**Figure 8:**
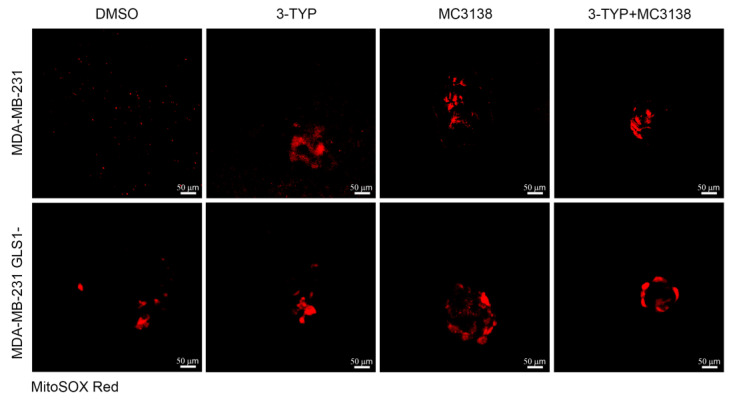
Increased mitochondrial ROS in spheroids treated with 3-TYP and MC3138. Spheroids were either treated with DMSO or with 3-TYP, MC3138 or 3-TYP plus MC3138 for 48 h. At the end of the treatments, spheroids were incubated with the MitoSOX probe (red fluorescence) to measure mitochondrial ROS as described under Materials and Methods. It should be noted that there is an increase in mitochondrial ROS staining in the spheroids from MDA-MB-231 GLS1- compared to the spheroids from wt cells, and an increase after the treatments in spheroids of both wt and GLS1- cells.

The effects of the accumulation of dysfunctional mitochondria were also investigated by measuring the mitochondrial specific ROS levels using the MitoSOX probe. Compared to DMSO-treated wt MDA-MB-231 cells, 3-TYP, MC3138 and 3-TYP+MC3138 treatments increased the amount of mitochondrial ROS (Figure 8).

Similar results were obtained in MDA-MB-231 GLS1- cells in which, however, we noticed a higher level of mitochondrial ROS also in DMSO control, confirming the important antioxidant role of glutamine metabolism (Figure 8 lower panel). Finally, mitochondrial ROS accumulation was determined after treating spheroids with BTES to inhibit GLS1 with or without 3-TYP plus MC3138 (Figure S7). Compared to DMSO, mitochondrial ROS increased in spheroids treated with BPTES and in BPTES plus 3-TYP+MC3138 where there were a strong ROS accumulation and a decrease in the size of the spheroid (Figure S7), resembling what was seen in GLS1- spheroids.

## 3. Discussion

Metabolic reprogramming is a hallmark of tumors [34]. Our previous work and the work of others have demonstrated the central role of glutamine and glutamine metabolism for cancer cells’ growth, survival and progression to a point that cancer cells become glutamine-addicted [4,15,35]. For this reason, a strategy that can re-establish the correct glutamine metabolism could have also anti-cancer properties without affecting the survival of non-cancer cells. In an attempt to address this hypothesis, the present work employed spheroids of TNBC MDA-MB-231 cells treated with SIRT3 and SIRT5 modulators. Our results show that a combination of a SIRT3 inhibitor and a SIRT5 activator was particularly effective in reducing the growth of spheroids (Figure 1). This particular combination was chosen due to the previous literature reporting; on the one hand, SIRT3 overexpression in breast cancer has been correlated with worse overall survival [36], and, on the other hand, a direct desuccinylation and inhibition of GLS by SIRT5 has been found [37,38]. In addition, SIRT3 is also involved in the same glutamine metabolism pathway by activating the second enzyme of the pathway, i.e., GDH. Considering these data, we reasoned that the best combination for reprogramming glutamine metabolism in TNBC cell line MDA-MB-231 was through SIRT5 activation and SIRT3 inhibition. In fact, using a combination of our validated SIRT5 activator MC3138 [15,26] and the widely used SIRT3 inhibitor 3-TYP [25,39] resulted in a decreased growth and clones formation in a 2D cell culture, as well as in a decreased growth of a 3D model (spheroids) (Figures S3, S4 and Figure 1). Importantly, both MC3138 and 3-TYP had no effect on primary fibroblasts (Figure S5). The important role of glutamine metabolism for the growth of spheroids was confirmed by the fact that MDA-MB-231 cells silenced for GLS formed smaller spheroids (Figure 1 and Figure 3).

An aspect worth noting reported in Figure 1B is our data regarding the global acetylation and succinylation level after treatments with 3-TYP, MC3138 or 3-TYP+MC3138. Our results show an increase in acetylated lysines in the presence of the SIRT5 activator MC3138. The crosstalk between different post translational modifications (PTMs) has been observed in yeast, mammalian cells, bacteria and developing rice seeds [40,41,42]. For example, it has been shown that p53 phosphorylation of Thr377 and Ser378 reduces acetylation of Lys373 and 382 [43]. Similarly, acetylation of Tau at Lys280 increased phosphorylation of Ser202 and 422 and Thr205 and 231 [44]. Finally, an overlap between succinylation and acetylation has been documented in prokaryotes (*E. coli* and yeast) and eukaryotes (HeLa and mouse liver). In fact, authors found that 66% of *E. coli*, 56% of yeast, 27% of human and 57% of mouse succinylation sites were acetylated at the same position [40]. Therefore, our results confirm in breast cancer cells what is also seen by others, and may suggest that the activators and inhibitors we used can be important tools to study the interplay among different PTMs.

Next, we investigated the molecular effects of our treatment. Tampering with the activity of the mitochondrial sirtuins SIRT3 and SIRT5 reduced autophagy and mitophagy markers LC3 and BNIP3 while increasing mitochondrial ROS (Figure 2, Figure 3 and Figure 8). In particular, our results suggest a scenario where damaged and dysfunctional mitochondria are not removed because of the reduction in autophagy and mitophagy proteins. In this scenario, such dysfunctional mitochondria represent the major source of ROS that accumulate in the treated spheroid, leading to cell death and spheroid disaggregation (Figure 1, Figure 2 and Figure 8). The reduction in the autophagy marker LC3 is linked to the reduction in ammonia normally produced during glutamine metabolism. In fact, low concentrations of ammonia have been shown to activate the autophagic pathway [45]. Cancer cells, where the glutamine metabolism is over-activated, use ammonia-induced autophagy as a protective mechanism. Moreover, ammonia can diffuse among neighboring tumor and non-tumor cells in the microenvironment, activating autophagy [46]. Interestingly, spheroid of MDA-MB-231 silenced for GLS showed a basal reduction in LC3 II that was not influenced by our treatment, confirming the important role of glutamine metabolism for autophagy induction (Figure 3). On the other hand, the mitophagy marker BNIP3 showed a decrease in spheroids treated with MC3138 or 3-TYP+MC3138 regardless of GLS silencing (Figure 2 and Figure 3). To investigate these results, we measured the expression of HIF-1α, a transcription factor activated by reduced oxygen tension, overexpressed in many tumors and responsible for Bnip3 gene transcription as well as autophagy and mitophagy activation [33]. Moreover, our 3D model of spheroids re-creates a hypoxic microenvironment at the center of the spheroid, increasing HIF-1α level. Interestingly, our results show a reduction in HIF-1α expression in the presence of MC3138 or 3-TYP+MC3138 (Figure 5). Such a reduction is due to SIRT5 activation and confirms what has been previously documented by us regarding cancer cells under hypoxia conditions treated with MC3138 [15]. Considering that, at the moment, there are no data demonstrating a direct succinylation of HIF-1α, we can only conclude that the decrease that we see with MC3138 is indirect. This can be explained considering that, in cancer cells, HIF-1α is stabilized by accumulating succinate through the inhibition of prolyl hydroxylase (PHD) [47]. SIRT5 by consuming succinyl-CoA reduces succinate and, indirectly, HIF-1α. On the other hand, we did not observe a reduction in HIF-1α with 3-TYP. This is because SIRT3 controls HIF-1α downregulation both directly through deacetylation and indirectly through ROS reduction [48,49]. Importantly, the reduction in HIF-1α correlates with the reduction in BNIP3 and mitophagy that, in fact, is obtained only when spheroids are treated with MC3138 or 3-TYP+MC3138 (Figure 2 and Figure 3). Another interesting observation is the low HIF-1α expression in GLS-silenced spheroids, suggesting a connection between glutamine metabolism and HIF-1α expression. We did not explore this aspect; however, HIF-1α is known to regulate both GLS and GLUL (glutamate-ammonia ligase) expression depending on the glutamine and glutamate levels [50] and, therefore, it is possible that altering glutamine metabolism can, conversely, regulate HIF-1α expression. In conclusion, even if the exact mechanism must be determined, our results indicate that SIRT5 activation through MC3138 decreases, either directly or indirectly, HIF-1α and, in turn, BNIP3. BNIP3 reduction prevents the removal of dysfunctional mitochondria, allowing for ROS accumulation and cell death. The anti-tumor effects of MC3138 and 3-TYP are extended also to c-Myc, a transcription factor with a central role in glutamine metabolism and often overexpressed in cancer cells [51]. c-Myc regulates expression of enzymes of the glutamine pathway such as GLS and glutamine transporters [30,52]. Our results show a reduction in c-Myc expression after 3-TYP and MC3138 treatment both in wt and GLS-silenced spheroids (Figure 5). To our knowledge, this is the first study linking SIRT3 inhibition and SIRT5 activation to c-Myc. Therefore, we can only hypothesize that the inhibition of c-Myc expression that we see could be due to direct effects on the acetylation/succinylation status of this protein, indirect effects on transcriptional partners such as MAX, or finally, on the energetic status of the treated cells. Importantly, the similar results observed with GLS1- cells were also obtained using the GLS1 inhibitor BPTES, in which an effect on BNIP3, LC3, HIF-1α and c-Myc are significant only in the presence of 3-TYP plus MC3138, indicating the important role of these sirtuins beyond their effect on glutamine metabolism (Figure S6). In addition, we also documented a reduction in the glutamine transporter SLC1A5 during the 3-TYP plus MC3138 treatment (Figure 5). Overall, our results indicate that a treatment obtained by combining SIRT3 inhibition and SIRT5 activation reduces or regulates three major aspects known to be up-regulated in TNBC cells: glutamine metabolism, HIF-1α and c-Myc expression and autophagy/mitophagy.

The effects of the molecular changes described above were investigated from a morphological and ultrastructural level. TEM analysis of untreated or treated spheroids revealed changes in mitochondria. In fact, 3-TYP and MC3138 treatments either alone or in combination shifted mitochondria to a condensed state from the orthodox one observed in the control. Interestingly, mitochondria in the condensed state have been associated with increased oxidative phosphorylation and ROS production [27]. Moreover, 3-TYP+MC3138 treatment increased cellular damage (Figure 4). Interestingly, spheroids from GLS-silenced cells presented mitochondria in the intermediate orthodox-condensed state that shifted to a condensed ROS-producing state after the treatments, confirming the important role of SIRT3, SIRT5 and glutamine for mitochondria state and function (Figure 4). The density of the mitochondria increased in cells of the wt spheroids following the treatments, an effect also due to the reduction in mitophagy (Figure 3 and Figure 4J). Interestingly, cells in GLS1- spheroids had a significant increase in mitochondrial density compared to the wt counterpart. However, this increase was not affected by the treatments, probably because of the increased cell damage (Figure 4J). The length of the mitochondria was higher in the wt than in the GLS1- spheroids (Figure 4I). Interestingly, there was no change in mitochondrial length in the GLS1- spheroids, even in the presence of the different treatments (Figure 4I), suggesting a role for glutamine metabolism in regulating mitochondrial size. Finally, we also documented the reduced lysosomes–mitochondria co-localization in spheroids of wt and GLS1- MDA-MB-231 cells following the 3-TYP+MC3138 treatment (Figure 6 and Figure 7). Finally, the same treatment strongly increased mitochondrial ROS production, confirming the presence of dysfunctional mitochondria (Figure S7 and Figure 8). The results of the present study also suggest that an inhibition of ROS accumulation using N-acetylcysteine or an induction of autophagy using rapamycin could restore spheroids’ viability upon 3-TYP plus MC3138 treatment. In fact, this has been already documented in other studies using 3D cellular systems, where N-acetylcysteine or rapamycin restored cellular viability through ROS scavenging or autophagy induction [53,54]. Even though we did not observe a toxic effect of our treatments on primary skin fibroblasts (Figure S5) or, in the case of MC3138, on a mouse model of pancreatic cancer [55], we cannot exclude the possibility of them having systemic adverse effects, considering the widespread and important role of sirtuins in physiology. This underlines the importance of translating our results in animal models. On the other hand, our study provides, for the first time, results on the effects of a combined treatment with PTM modulators, a strategy recently also suggested by other groups [36,56,57]. In addition, one can also foresee a combination of our treatment with standard anti-tumoral treatments such as doxorubicin, cisplatin or immunotherapy.

## 4. Materials and Methods

### 4.1. Cell Culture and Treatments

The MDA-MB-231 human breast carcinoma cell line was obtained from LGC Standards (ATCC-HTB-26, Milan, Italy) and was grown in RPMI1640 medium (MERCK, St. Louis, MO, USA. R0883) with supplementation of 10% Fetal Bovine Serum (MERCK; F9665), 2 mM Glutamine (MERCK; G7513), 100 units/mL penicillin and 0.1 mg/mL streptomycin (MERCK; P0781). Primary fibroblasts were isolated from skin biopsies. Skin biopsies were cut into small pieces, placed in a well of a six-well plate filled with DMEM plus 10% FBS and covered with a glass coverslip. After 15–20 days, the growing fibroblasts were detached by Trypsin-EDTA solution (MERCK; T4049) collected and re-plated in a six-well plate for the experiments. MDA-MB-231 cells were detached by Trypsin-EDTA solution (MERCK; T4049). All cell lines were maintained at 37 °C in a humidified atmosphere of 5% CO_2_ and 95% air. MDA-MB-231 was stably transfected using a pLKO.1 vector containing a shRNA insert targeting human GLS1 (MERCK; SHCLND-NM 014905) [15].

Cells were treated with 25 nM of SIRT3 inhibitor 3-TYP [58], 50 μM of SIRT3 activator MC2791 and BPTES 10 μM (MERCK) dissolved in DMSO for 48h. Even though 3-TYP is commercially available, the one used by us was synthesized, along with SIRT3 and SIRT5 modulators, by the laboratory of Professor Mai of the Department of Chemistry and Drug Technologies at Sapienza University of Rome. The synthesis, characterization and validation of MC3138 and 3-TYP has been previously described [15,26,51]. Control cells were treated with the same concentration of DMSO used to dissolve the compounds and the results were normalized to the concentration. Each experiment described below was repeated three times.

### 4.2. Trypan Blue Assay

Cells were seeded in a 100 mm dish and, once at 80–90% confluence, treated as described. After 24 and 48 h, cells were collected and diluted 1:5 with Trypan Blue. The cell suspension was applied to a hemocytometer and counted with a phase contrast microscopy (NIKON Eclipse TE 2000U, Nikon Netherlands, Amsterdam, The Netherlands).

### 4.3. Clonogenicity Assay

Cells were seeded in a 100 mm dish and, once at 80–90% confluence, treated as described. After 24 and 48 h, cells were collected, counted and 500 plated in a 100 mm dish. After about 10 days, plates were washed with phosphate-buffered saline solution (PBS; MERCK; 79382) and clones fixed with 4% formaldehyde solution in PBS (MERCK; F8775) at rt for 15 min. After that, the dishes were washed with PBS and clones stained for 5 min with 0.5% crystal violet (MERCK; C0775). Finally, plates were washed with distilled water and air-dried. After scanning each individual dish, the colonies were counted the following day.

### 4.4. Spheroids Generation

Spheroids were generated by plating 1 × 10^3^ cells in a six-well ultra-low attachment plate for immunofluorescence and electron microscopy studies and 15 × 10^4^ cells in T75 ultra-low attachment flasks for Western blot assays. Spheroids were grown for 7 days before starting the treatments. Spheroids’ size was measured considering the diameter of at least five spheroids. Fluorescent analysis of the spheroids was performed by observing three to five spheroids on the coverslip.

### 4.5. Proteins Extraction and Immunoblotting

Treated spheroids were collected and centrifuged at 1500 rpm for 5 min. Spheroids were lysed in 25 μL of lysis buffer containing 50 mM Tris-Cl (MERCK; 93352), 250 mM sodium chloride (NaCl, MERCK; S7653), 5 mM ethylenediaminetetraacetic acid (EDTA; MERCK; E6758), 0.1% Triton^®^ X-100 and 0.1mM Dithiothreitol (DTT, MERCK; D9163) plus 1 mM phenylmethylsulfonyl fluoride (PMSF, MERCK; 93482), Protease inhibitor cocktail (PI; MERCK; P8340), 1 mM sodium orthovanadate (NA_3_VO_4_, MERCK; S6508) and 10 mM sodium fluoride (NaF, MERCK;201154) (lysis buffer) and left in ice for 30 min. After the samples were centrifuged at 13,000 rpm for min at 4 °C and the supernatants collected. Protein concentration was determined by the Bradford assay (Bio-Rad, Milan, Italy; 500-0205). An equivalent amount of proteins was boiled for 5 min, electrophoresed onto denaturating SDS-PAGE gel and transferred onto a 0.45 μM nitrocellulose membrane (Bio-Rad; 162-0115). Then, membranes were blocked with 5% milk for 1 h and incubated with the appropriate primary antibody overnight. The next day, after three washes with 0.1% Tween^®^ 20 (MERCK; P9416) in PBS (PBST) at room temperature, membranes were incubated with the appropriate secondary antibody for 1h at room temperature, followed by three washes, after which the detection of the relevant protein was assessed by enhanced chemiluminescence (LiteAblot^®^ TURBO, EuroClone, Milan, Italy; EMP012001). The following antibodies were used in this study: Acetylated-Lysine (Cell Signaling, Milan, Italy; #9441), BNIP3 (Santa Cruz Biotechnology, Heidelberg

Germany; sc-56167), Succinyl-Lysine (PTM BIO, Milan, Italy; PTM-419), GAPDH (Santa Cruz Biotechnology; sc-137179), HIF-1α (Cell Signaling; 14179), c-Myc (Thermo Fisher Scientific, Waltham, MA, USA, 10828-1-AP), LC3 (Novus Biologicals, Mila Italy; NB600-1384), Peroxidase-conjugated AffiniPure Goat Anti-Rabbit IgG (H+L) (Jackson ImmunoResearch, Milan, Italy; 111-035-003), Peroxidase-conjugated AffiniPure Goat Anti-Mouse IgG (H+L) (Jackson ImmunoResearch; 115-035-062). Protein content in the membrane was visualized by using the ChemiDoc TM MP Imaging System (Bio-Rad). Densitometric analysis of the bands, relative to GAPDH, was performed using ImageJ Software v1.53 (NIH, Bethesda, MD, USA).

### 4.6. Immunofluorescence

After 48 h of treatment, spheroids were collected and centrifugated at 300 rpm for 3 min. They were washed three times with PBS (MERCK, D8537) and were fixed with 4% paraformaldehyde (Immunofix, Bio-optica, Milan, Italy; 05-K01015) and 0.5% glutaraldehyde (Sigma-Aldrich-MERCK, St. Louis, MO, USA, G7651) for 24 h at 4 °C. The day after, fixed spheroids were washed in PBS and incubated for 4 h at room temperature with an anti-BNIP3 (1:500) or anti-LC3 (1:1000). After three washes, the spheroids were incubated for 1 h with a goat anti-rabbit and a goat anti-mouse IgG Alexa Fluor 555 fluorescent secondary antibody (1:1000, Invitrogen, Carlsbad, CA, USA). Then, three washes were carried out, and nuclei were stained by incubating the spheroids for 8 min with SYTO 16 green fluorescent nucleic acid stain 1:30,000 (Invitrogen, Eugene, OR, USA, S7578). Finally, spheroids were washed with PBS three times, mounted in ProLong Diamond Antifade Mountant (Life Technologies, Thermo Fisher Scientific, 196 Carlsbad, CA, USA—P36961) and analyzed with a LSM510 confocal microscope (Zeiss, Oberkochen, Germany).

### 4.7. Organelles Staining

Lysotracker^®^ red DND-99 (L7528; Thermo Fisher—Life Technologies, Waltham, MA, USA) was used to highlight cells lysosomes in spheroids. After 48 h of treatment with SIRT5 activator and SIRT3 inhibitor (individually and/or in combination), spheroids were washed twice with PBS and incubated for 10 min with culture medium, in which the Lysotracker was dissolved at a concentration of 5 µM. Subsequently, mitochondria were stained with Mitotracker™ Green FM (M7514; Thermo Fisher—Life Technologies) for 20 min at concentration of 10 nM. Finally, spheroids were washed twice with PBS and placed on a slide. Fluorescence was observed with a confocal microscope LSM 510 (Zeiss, Oberkochen, Germany).

### 4.8. Reactive Oxygen Species Staining

MitoSOX Red (mitochondrial superoxide indicator, Invitrogen, Life Technologies Corporation, Eugene, OR, USA) at concentration of 1 mM for 30 min was used to assess levels of mitochondria ROS (reactive oxygen species) in spheroid cells treated for 48 h with SIRT5 activator and SIRT3 inhibitor (individually and/or in combination). MitoSOX was dissolved in the culture medium and subsequently the spheroids were washed twice and placed on a slide. ROS levels were assessed by confocal fluorescence (LSM510, Zeiss, Oberkochen, Germany).

### 4.9. TEM

Spheroids were collected and fixed overnight in 2.5% glutaraldehyde (prepared in 0.1 M phosphate buffer, pH 7.2). Afterward, the samples were rinsed six times in phosphate buffer and post-fixed in 2% osmium tetroxide (also in 0.1 M phosphate buffer) for 2 h at room temperature. They were then processed according to a standard protocol for EPON embedding. After overnight polymerization at 65 °C, sections (70 nm) were cut using a Righter-Jung Ultra cut E Ultramicrotome (Leica Microsystems, Wetzlar, Germany) and mounted on copper grids. The sections were stained with a uranyl substitute and lead hydroxide before being examined with a JEOL TEM-1400 Plus transmission electron microscope (JEOL, Milan, Italy) [59].

### 4.10. Morphometric Analysis

Mitochondria length and numerical density were evaluated as previously reported [44]. Briefly, at least 15 specimens per group were imaged using TEM at the same magnification (2500×) on at least five equatorial sections (distance between the sections: 3–4 µm) and analyzed by means of ImageJ 1.53 software. The digital images were additionally magnified to facilitate the identification and enumeration of the organelles. Mitochondrial numerical densities are expressed as density per cell.

### 4.11. Statistical Analysis

All data were expressed as means ± standard deviation (SD). Statistical comparisons for TEM analysis were performed using one-way ANOVA with Tukey honest significant difference (HSD) tests for post hoc analysis (GraphPad InStat 10.1.2. GraphPad Software, San Diego, CA, USA). Otherwise, statistical analysis between pairs of groups was performed by Student’s *t*-test. Differences in values were considered significant if *p* < 0.05.

## 5. Conclusions

In conclusion, our results show that a combined treatment with SIRT3 and SIRT5 modulators has both direct and indirect anti-tumor effects. Directly by inhibiting the growth of TNBC spheroids, by increasing global acetylation and decreasing succinylation and by altering glutamine metabolism. Indirectly through a mechanism involving HIF-1α inhibition, autophagy and mitophagy inhibition and mitochondrial ROS production.

## Data Availability

The original contributions presented in this study are included in the article/Supplementary Material. Further inquiries can be directed to the corresponding authors.

## Supplementary Materials

The following supporting information can be downloaded at https://www.mdpi.com/article/10.3390/ph19010023/s1, Figure S1: Effects of SIRT3 inhibition and SIRT5 activation on wt and GLS1- MDA-MB-231 cells; Figure S2: Effects of SIRT3 inhibition and SIRT5 activation on clones formation of wt and GLS1- MDA-MB-231 cells; Figure S3: Effects of SIRT3 inhibition and SIRT5 activation on wt and GLS1-MDA-MB-231 cells; Figure S4: Effects of SIRT3 inhibition and SIRT5 activation on clones formation of wt and GLS1-MDA-MB-231 cells; Figure S5: Effects of SIRT3 inhibition and SIRT5 activation on primary fibroblasts; Figure S6: Effects of BPTES on BNIP3, LC3, HIF-1α and c-Myc expression; Figure S7: Effects of BPTES on mitochondrial ROS accumulation in spheroids from MDA-MB-231.
