# Supplementary material for: A Combined SIRT5 Activation and SIRT3 Inhibition Prevents Breast Cancer Spheroids Growth by Reducing HIF-1α and Mitophagy
Source: Pharmaceuticals (Basel). 2025 Dec 22;19(1):23. doi: 10.3390/ph19010023 (PMC12844701; doi:10.3390/ph19010023)
Supplement: Supplementary file 1 [file pharmaceuticals-19-00023-s001.zip › pharmaceuticals-3959431-supplementary.pdf]

A

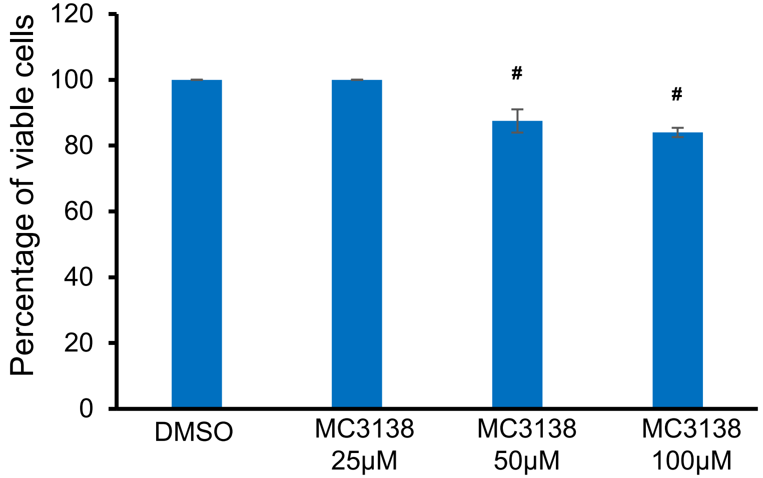

B

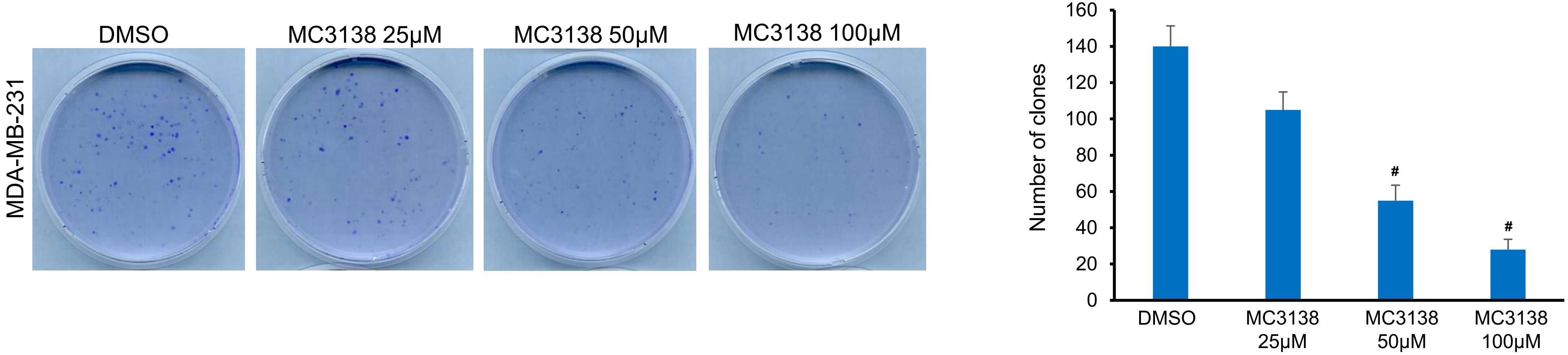

Figure S1. Cell viability and clonogenicity after treatment of MDA-MB-231 cells with increasing doses of MC3138
An equal number of wt MDA-MB-231 cells was plated in 100mm dishes. The day after the cells were either left untreated or treated increasing concentrations of MC3138 for 48h. (A) The percentage of viable cells was determined by Trypan blue exclusion.(B) After 7 days the clones were fixed, stained and counted. Experiment was repeated three times. * Significantly different from DMSO treated cells. * $p < 0.05$.

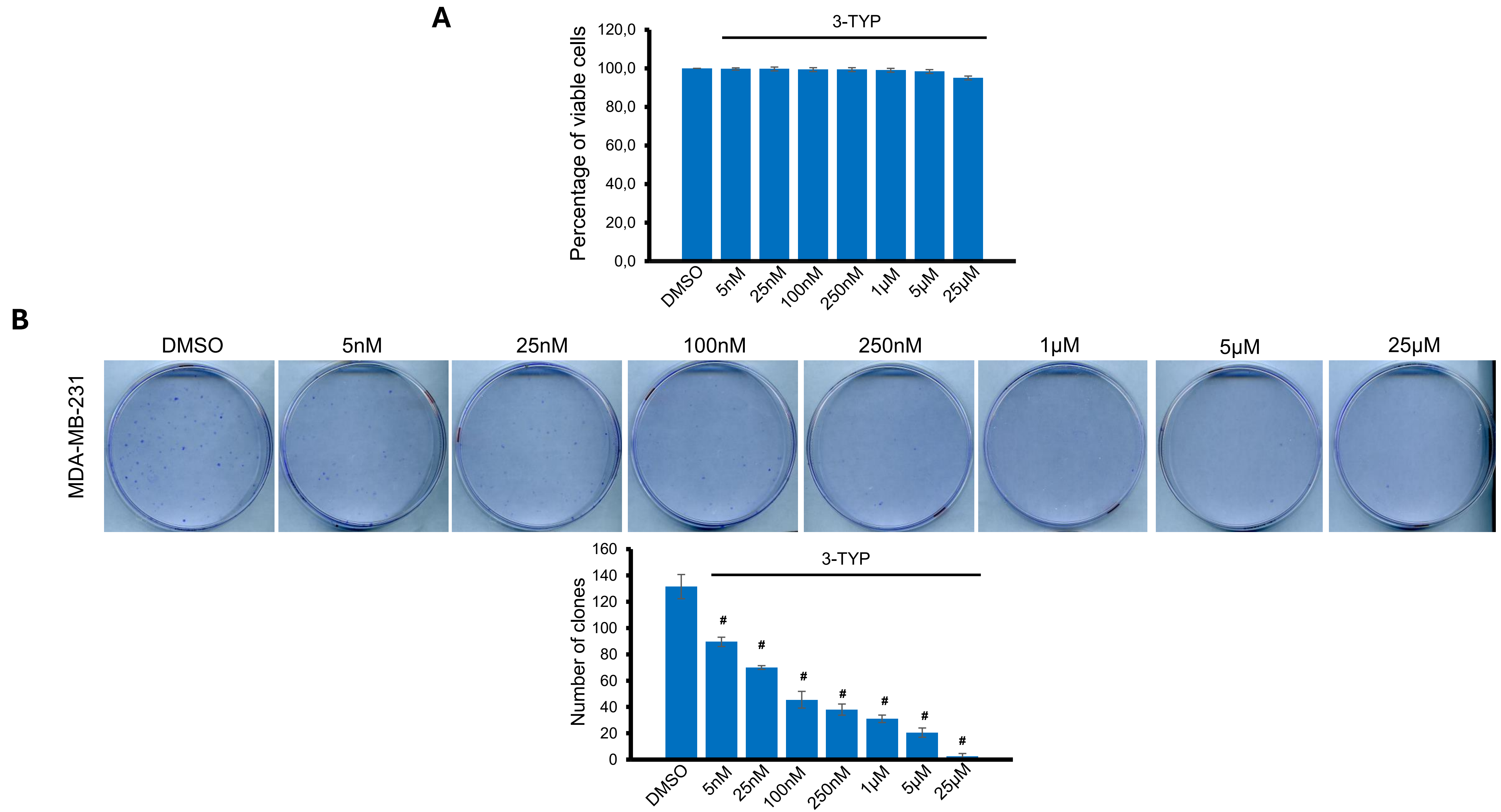

Figure S2. Cell viability and clonogenicity after treatment of MDA-MB-231 cells with increasing doses of 3-TYP

MDA-MB-231 cells were treated with increasing concentrations of 3-TYP for 48h. At the end of the treatment cells were counted and an equal number plated in 100mm dishes. (A) The percentage of viable cells was determined by Trypan blue exclusion. (B) After 7 days the clones were fixed, stained and counted. Images are representative of three separate experiments. * Significantly different from DMSO treated cells. * $p < 0.05$.

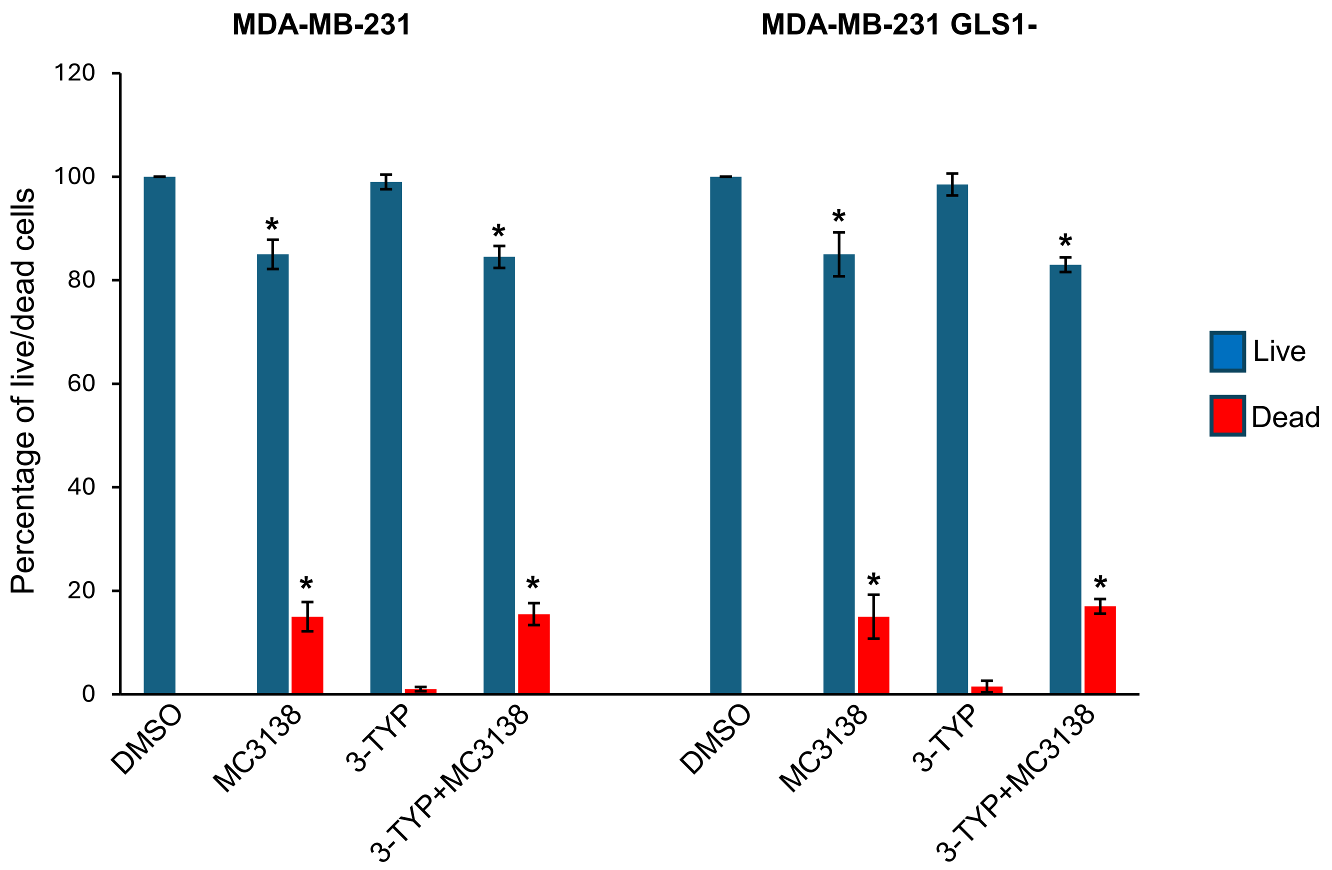

Figure S3. Effects of SIRT3 inhibition and SIRT5 activation on wt and GLS1- MDA-MB-231 cells.

An equal number of wt and GLS1- MDA-MB-231 cells was plated in 100mm dishes. The day after the cells were either treated with DMSO or as described in Materials and Methods. The percentage of live and dead cells was determined by Trypan blue exclusion. Experiment was repeated three times. * Significantly different from DMSO treated cells. * $p < 0.05$.

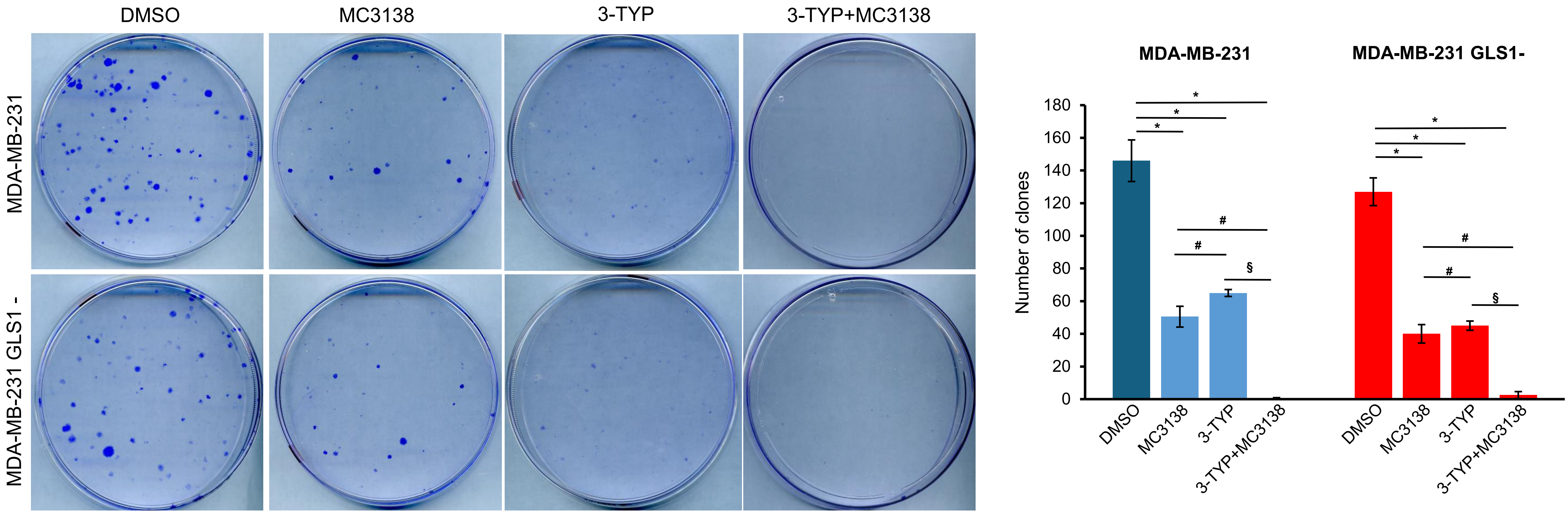

Figure S4. Effects of SIRT3 inhibition and SIRT5 activation on clones formation of wt and GLS1- MDA-MB-231 cells.
Wt and GLS- MDA-MB-231 cells were treated with 3-TYP, MC3138 or 3-TYP plus MC3138 for 48h. At the end of the treatment cells were counted and an equal number plated in 100mm dishes. After 7 days the clones were fixed, stained and counted. Images are representative of three separate experiments. * Significantly different from DMSO treated cells. #, Significantly different from 3-TYP and 3-TYP plus MC3138 treatment. §, Significantly different from 3-TYP plus MC3138 treatment. Significance was set at $p < 0.05$.

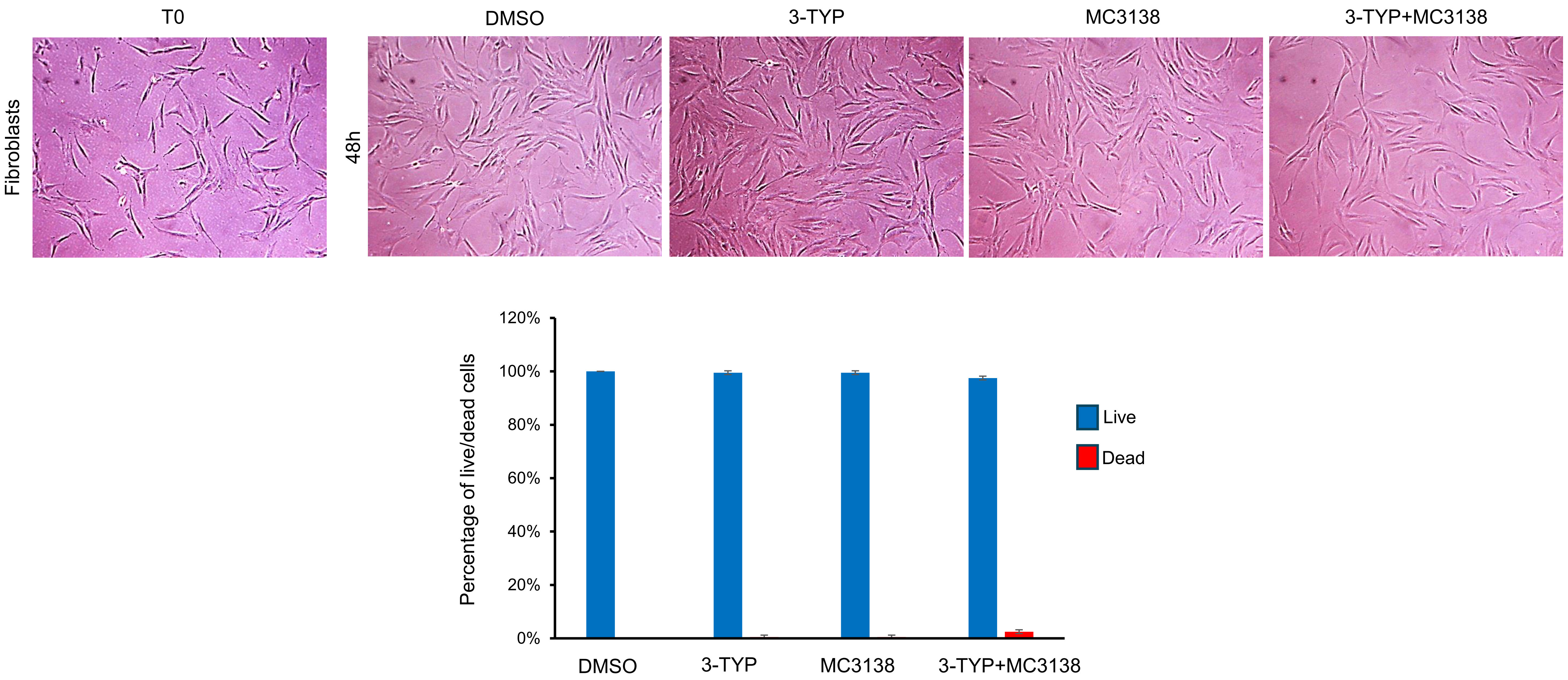

Figure S5. Effects of SIRT3 inhibition and SIRT5 activation on primary fibroblasts.
An equal number fibroblasts were plated on a 6 wells plate. The day after the cells were either left untreated or with 3-TYP (25nM), MC3138 (50μM) or 3-TYP plus MC3138 for 48h. The percentage of live and dead cells was determined by Trypan blue exclusion. Experiment was repeated three times.

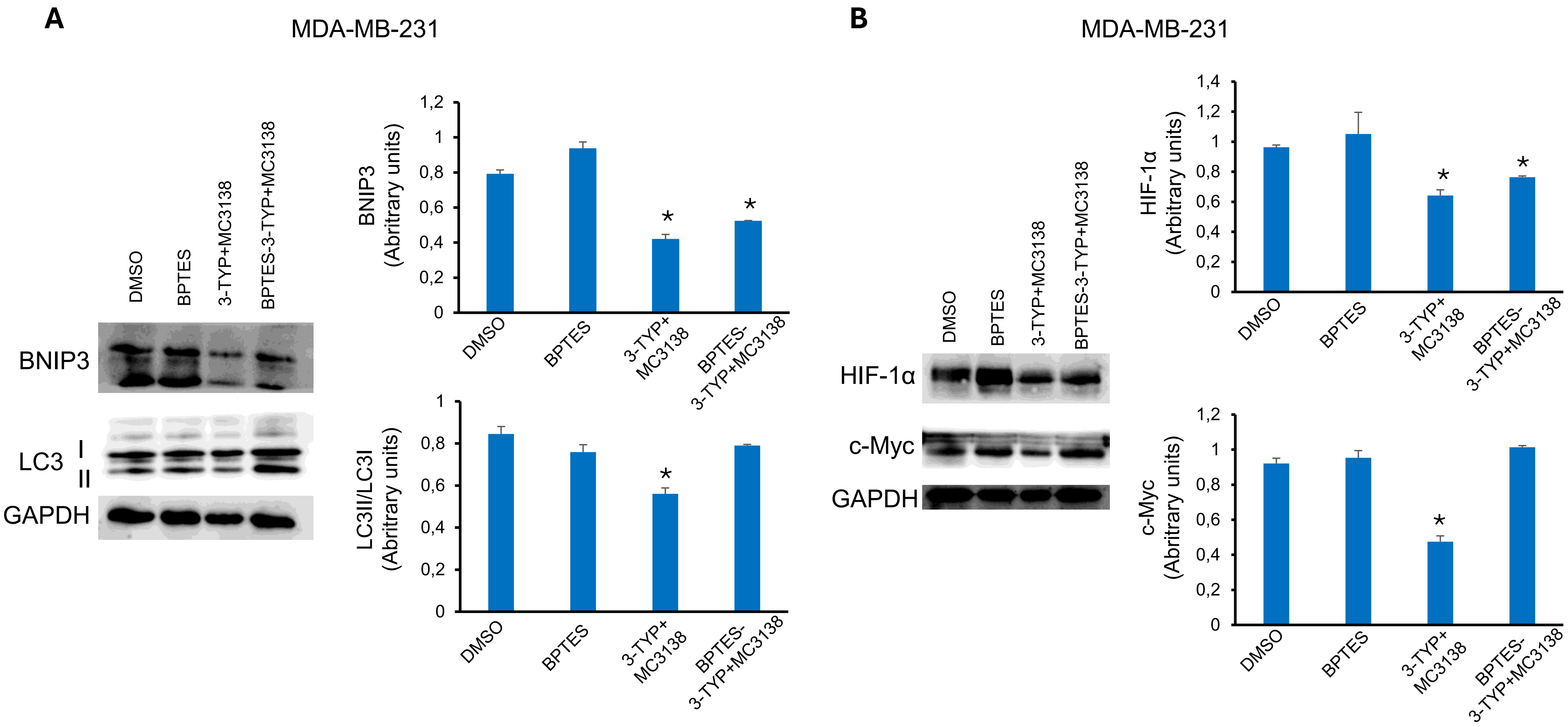

Figure S6. Effects of BPTES on BNIP3, LC3, HIF-1α and c-Myc expression.
Spheroids were either treated with DMSO or with BPTES (10µM) or 3-TYP(25nM)+MC3138 (50µM) or BPTES+3-TYP+MC3138 for 48h. Expression of BNIP3 and LC3 (A) or HIF-1α and c-Myc (B) was measured by western blot as described in Materials and Methods. Experiment was repeated three times. * Significantly different from DMSO treated cells. * $p < 0.05$.

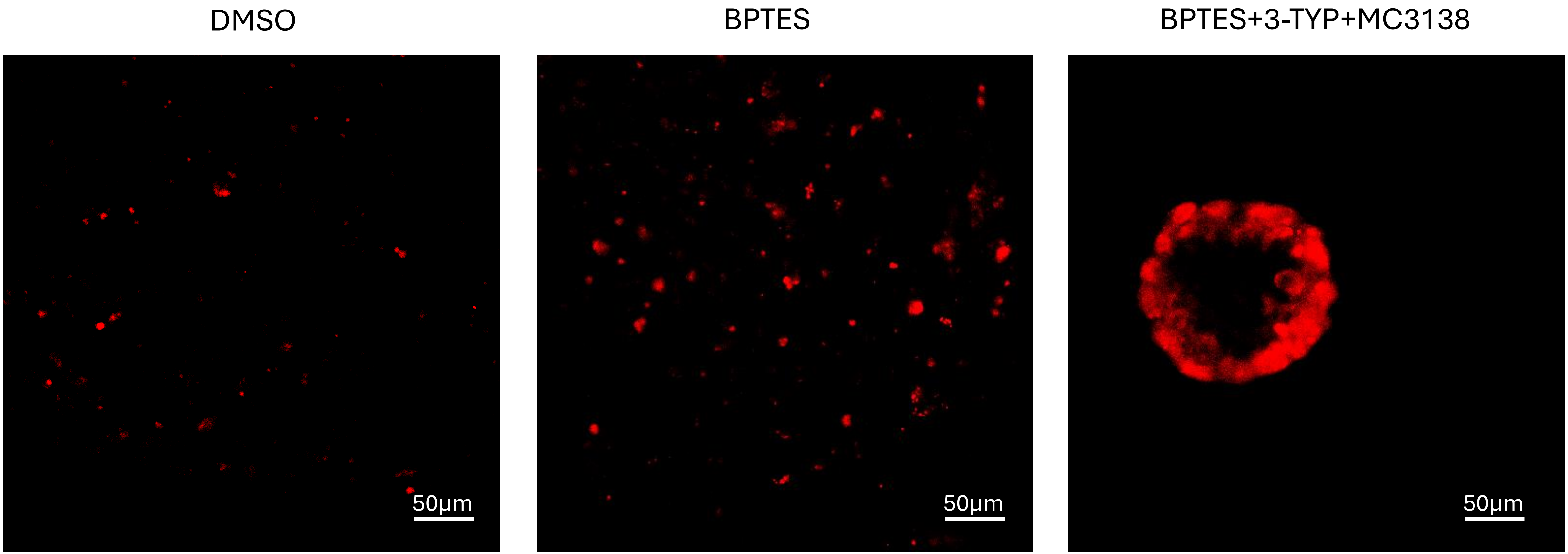

Figure S7. Effects of BPTES on mitochondrial ROS accumulation in spheroids from MDA-MB-231.
Spheroids were either treated with DMSO or with BPTES (10μM) or BPTES+3-TYP(25nM)+MC3138 (50μM) for 48h. At the end of the treatments, spheroids were incubated with the MitoSOX probe to measure mitochondrial ROS as described under Materials and Methods. To note the increase mitochondrial ROS staining and the decrease in size of the treated spheroids compared to the DMSO ones.
